# Impact of miR-181a on SIRT1 Expression and Senescence in Hutchinson–Gilford Progeria Syndrome

**DOI:** 10.3390/diseases13080245

**Published:** 2025-08-04

**Authors:** Eva-Maria Lederer, Felix Quirin Fenzl, Peter Krüger, Moritz Schroll, Ramona Hartinger, Karima Djabali

**Affiliations:** Epigenetics of Aging, Department of Dermatology and Allergy, TUM School of Medicine, Munich Institute of Biomedical Engineering (MIBE), Technical University of Munich (TUM), 85748 Garching, Germany; eva.lederer@tum.de (E.-M.L.);

**Keywords:** HGPS, aging, miR-181a-5p, SIRT1, TGFβ1, senescence

## Abstract

Background/Objectives: Hutchinson–Gilford progeria syndrome (HGPS) is a rare and fatal genetic disease caused by a silent mutation in the LMNA gene, leading to the production of progerin, a defective prelamin A variant. Progerin accumulation disrupts nuclear integrity, alters chromatin organization, and drives systemic cellular dysfunction. While autophagy and inflammation are key dysregulated pathways in HGPS, the role of microRNAs (miRNAs) in these processes remains poorly understood. Methods: We performed an extensive literature review to identify miRNAs involved in autophagy and inflammation. Through stem-loop RT-qPCR in aging HGPS and control fibroblast strains, we identified significant miRNAs and focused on the most prominent one, miR-181a-5p, for in-depth analysis. We validated our in vitro findings with miRNA expression studies in skin biopsies from an HGPS mouse model and conducted functional assays in human fibroblasts, including immunofluorescence staining, β-Galactosidase assay, qPCR, and Western blot analysis. Transfection studies were performed using an miR-181a-5p mimic and its inhibitor. Results: We identified miR-181a-5p as a critical regulator of premature senescence in HGPS. miR-181a-5p was significantly upregulated in HGPS fibroblasts and an HGPS mouse model, correlating with Sirtuin 1 (SIRT1) suppression and induction of senescence. Additionally, we demonstrated that TGFβ1 induced miR-181a-5p expression, linking inflammation to miRNA-mediated senescence. Inhibiting miR-181a-5p restored SIRT1 levels, increased proliferation, and alleviated senescence in HGPS fibroblasts, supporting its functional relevance in disease progression. Conclusions: These findings highlight the important role of miR-181a-5p in premature aging and suggest its potential as a therapeutic target for modulating senescence in progeroid syndromes.

## 1. Introduction

Hutchinson–Gilford progeria syndrome (HGPS, OMIM 176670) is a rare premature aging disease caused primarily by a single de novo mutation at codon 608 in the *LMNA* gene (c.1824C>T, GGC>GCT). Despite being a silent mutation (G608G), it introduces a cryptic splice site, leading to an altered transcript that lacks a critical proteolytic cleavage site [1,2,3]. This mutation results in the production of a truncated, permanently farnesylated prelamin A variant called progerin. Progerin accumulates in cells, disrupting the nuclear lamina, a meshwork of type V intermediate filaments that provides structural support to the nucleus [4]. The abnormal retention of the farnesyl residue at the inner nuclear envelope leads to nuclear envelope dysfunction. These structural and nuclear defects are thought to drive the premature aging symptoms characteristic of HGPS patients, including low body weight, alopecia, skin lesions, atherosclerosis, and lipodystrophy [5]. Most HGPS patients succumb to myocardial infarction or stroke at an average age of 14.6 years [5,6]. While recent advances have led to the first FDA-approved treatment with lonafarnib, it is not a cure [7,8].

The HGPS phenotype arises from the dysregulation of multiple signaling pathways. Among these, mitochondrial dysfunction, characterized by excessive reactive oxygen species (ROS) production, impaired oxidative phosphorylation, and reduced ATP synthesis play an important role in premature cellular aging in HGPS [9,10]. In addition to mitochondrial impairment, alterations in glycolytic enzymes, induction of the DNA damage response, chronic inflammation, and defective autophagy have also been implicated in HGPS pathogenesis [10,11,12,13,14,15].

Autophagy, the primary cellular degradation pathway, eliminates aggregated and misfolded proteins, thereby maintaining proteostasis. Beyond its fundamental role in cellular homeostasis, autophagy is also implicated in neurodegenerative diseases, cancer, liver disease, and immune regulation, making it a key target for therapeutic intervention [16,17]. Inducing autophagy, e.g., by rapamycin or its analog Everolimus, enhances the clearance of toxic protein aggregates, as evidenced by reduced progerin levels and improved nuclear morphology [18,19,20]. Moreover, Everolimus has recently been tested in clinical trials together with lonafarnib (NCT02579044) [21]. Pharmacological activation of autophagy using AMP-activated protein kinase (AMPK) activators or ATP analogs to stimulate Unc-51-like kinase 1 (ULK1) has shown promise in preclinical models [19,22].

The innate immune response is the first line of defense against pathogens. Its complex inflammatory reaction network is interconnected and tightly regulated [23]. A text-mining approach used to identify the molecular signatures associated with four distinct HGPS-related pathologies—vascular disease, arthritis, alopecia, and lipodystrophy—revealed that proinflammatory cytokine-mediated activation of the Janus Kinase/Signal Transducer and Activator of Transcription (JAK1/2-STAT1/3) pathway plays a critical role in the development of these conditions [24]. Additionally, the nuclear factor kappa-light-chain-enhancer of activated B-cells (NF-κB) pathway is similarly implicated in HGPS pathologies, as its hyperactivation accelerates aging phenotypes in progeroid mice [25]. Importantly, pharmacological inhibition of NF-κB-mediated inflammation in endothelial cells significantly attenuates HGPS-related aging features [13,26].

MicroRNAs (miRNAs) play a critical role in numerous biological processes and in the pathogenesis of various diseases [27]. First discovered in 1993, miRNAs are short ~22 nt (19–25 nt) non-coding RNAs that regulate gene expression post-transcriptionally [28]. They exert their function by binding to target mRNAs through complementary base pairing, leading to mRNA degradation and translational repression. While perfect complementarity results in direct mRNA cleavage, partial complementarity inhibits translation without mRNA degradation [28,29,30]. Despite growing evidence of their biological significance, the role of miRNAs in HGPS remains largely unexplored. One exception is miR-9, a brain-specific miRNA that suppresses lamin A and progerin expression, which may contribute to the absence of cognitive decline in HGPS patients [31]. Additionally, the miR-29 family has been found to be upregulated in a progeroid mouse model, suggesting a link between miRNA dysregulation and aging phenotypes [32]. Other studies revealed the involvement of miR-1, miR-365, miR-376, miR-59, miR-34, and others in premature aging, generating beneficial or deleterious effects on the aging process [33,34,35,36].

In this study, we aim to identify additional miRNAs that are dysregulated in HGPS and to elucidate their functional roles. Our research focuses on miRNAs that play a significant role in the two critical cellular processes, both frequently disrupted in HGPS—inflammation and autophagy. We specifically investigated the highly upregulated miRNA miR-181a-5p and assessed its impact on the cellular defects observed in aged and HPGS cells.

## 2. Materials and Methods

### 2.1. Literature Review to Identify miRNA Implicated in the Regulation of Inflammation and Autophagy

We performed a text-mining procedure to identify critical miRNAs involved in the modulation of inflammation and autophagy, using PubMed and open-access databases. We targeted miRNAs functionally involved in these two key cellular processes that are highly relevant to HGPS pathology. For the autophagic pathway, we focused on miRNAs associated with critical proteins involved in the autophagic vesicles’ initiation, elongation, and fusion [37]. Given the vast number of proteins associated with inflammatory pathways, we narrowed our search to essential components of the Interleukin 6 signaling and canonical and non-canonical NF-κB signaling pathways, which are believed to be significantly affected in HGPS [13,24,26]. The PubMed search was conducted using keyword combinations such as “miRNA AND IL6R”, “miRNA AND ATG5”, and “miRNA AND RELA”, among others. Only studies reporting experimentally validated miRNA-target interactions, particularly in human cells, were considered. To further enhance specificity, we cross-validated these findings using open-access databases including miRTarBase (https://bio.tools/mirtarbase, 9 October 2024), TargetScan (https://www.targetscan.org/vert_80, 9 December 2024), and miRWalk (http://mirwalk.umm.uni-heidelberg.de, 9 December 2024). We retained only those miRNAs that were associated with both autophagy- and inflammation-related targets, based on the hypothesis that dual-pathway miRNAs may serve as central regulatory nodes relevant to HGPS.

### 2.2. Cell Culture

Control fibroblasts (GMO1651, GMO1652, GMO3349c, GMO1582B, GMO5565, GMO5757c, HGMDFN368, HGFDFN369) and HGPS fibroblasts (HGADFN127, HGADFN178, HGADFN003, HGADFN188, HGADFN271, HGADFN164) were obtained from the Coriell Institute for Medical Research (Camden, NJ, USA) and the Progeria Research Foundation Cell and Tissue Bank [38,39]. They were cultured in Dulbecco’s modified eagle medium (31966047, DMEM, Thermo Fisher/Gibco, Waltham, MA, USA/Grand Island, NY, USA) supplemented with 15% fetal bovine serum (10270106, FBS, Gibco), 1% L-glutamine (25030081, Thermo Fisher, Gibco), 1% Penicillin/Streptomycin (15140122, Thermo Fisher, Gibco), and 0.5% Gentamycin (15710049, Thermo Fisher, Gibco). Cultures were maintained in an incubator (Binder, Tuttlingen, Germany, 9140-0046) at 37 °C with 5% CO_2_. Cells were harvested or split upon reaching 80% confluency. In each passage, the senescence index was calculated to determine the actual age of the culture (see Senescence-Associated β-Galactosidase Staining). Table 1 lists the senescence values based on the passage number of the cell lines and the abbreviations used in the results section.

### 2.3. Drug and miRNA Mimic Transfection

As previously described in the literature [40], cells were cultured in Dulbecco’s modified eagle medium (DMEM, 10313021, Thermo Fisher/Gibco) supplemented with 10% FBS, 1% L-glutamine, 1% Penicillin/Streptomycin, and 0.5% Gentamycin and starved in the same medium with 0.5% FBS for 24 h before treatment. Cultures were treated in the starving medium with 10 ng/ml transforming growth factor β1 (TGFβ1, HZ-1011, Proteintech, Rosemont, IL, USA) for 96 h and collected for RNA isolation or fixed for immunofluorescence staining.

miR-181a-5p mimic (Thermo Fisher, miRVana™ miR-181a-5p mimic, 4464066) and miR-181a-5p inhibitor (Thermo Fisher, miRVana™ miR-181a-5p inhibitor, 4464084) were transfected in a concentration of 25 µM for 6 and 9 days and 10 nM for 6 and 9 days, respectively, at a cell density of 30–50%. As transfection control, miRVana™ miRNA inhibitor negative control number 1 (4464076, Thermo Fisher) and miRVana™ miRNA mimic negative control number 1 (4464058, Thermo Fisher) were transfected in the same concentrations. INTERFERIn^®^ (101000028, Polyplus, Illkirch, France,) was used as the transfection reagent according to the manufacturer’s protocol. The media and treatments were refreshed every third day.

### 2.4. Senescence-Associated β-Galactosidase Staining

The senescence index of the fibroblast cultures was determined using the protocol described by Dimri et al. [41]. Cells were washed with Dulbecco’s phosphate-buffered saline (PBS, D8537, Sigma-Aldrich, St. Louis, MI, USA), and fixed with a fixation buffer consisting of 2% Formaldehyde (104003, Sigma-Aldrich) and 0.2% Glutaraldehyde (G5882, Sigma-Aldrich) in PBS_._ After two steps of washing with PBS, staining was performed with freshly prepared buffer containing 5 mM Potassium Ferricyanide (III) (104973, Merck, Rahway, NJ, USA), 5 mM Potassium Ferrocyanide (II) (P9387, Sigma-Aldrich), 2 mM MgCl_2_ (M-1028, Sigma-Aldrich), 150 mM NaCl (310166, Sigma-Aldrich), and 0.5 mg/ml 5-bromo-4-chloro-3-indolyl P3-D-galactoside (3117073001, X-gal, Sigma-Aldrich,) in a 40 mM citrate/sodium phosphate buffer, pH 6.0, and incubated overnight at 37 °C without CO_2_. Blue-stained cells were counted as positive for senescence. We evaluated 1000 cells per culture to estimate the percentage of senescence in each passage. Table 1 shows the senescence indexes related to the passage numbers of the fibroblast strains used.

### 2.5. RNA Isolation and Quality Control

For each culture, cells were washed with PBS and detached with Trypsin (25200056, Thermo Fisher, Gibco). Following serum neutralization, cells were centrifuged at 1500 rpm for 5 min, and pellets were washed with PBS. Cell pellets were frozen at −80 °C or used directly. RNA was isolated using the miRNeasy Mini Kit (217004, Qiagen, Hilden, Germany) according to the manufacturer’s protocol. Concentration was measured by dissolving the sample in 1 mM Tris buffer, pH 7.0 (T1503, Sigma), using a Nanodrop ND-1000 spectrophotometer (Thermo Fisher). RNA was diluted in 1 mM Tris buffer, pH 7.5, for quality control, and the absorbance ratio at 260/280 nm was measured. Values between 1.9 and 2.1 indicate high-quality and intact RNA. RNA was aliquoted and stored at −80 °C for further use.

### 2.6. Reverse Transcription

cDNA synthesis of miRNAs was performed using the high-capacity cDNA reverse transcription kit (4368814, Thermo Fisher) employing stem-loop reverse transcription [42]. We designed RT stem-loop primers with 8 complementary nucleotides to ensure exact template amplification using snRNAprimerDB [43]. All primers were ordered at Thermo Fisher (10336022), and the sequences are cataloged in Table S1. The reverse transcription reactions were performed according to the manufacturer’s protocol using the following conditions: 30 min at 16 °C, followed by 60 cycles of 30 s at 30 °C, 30 s at 42 °C, and 1 s at 50 °C, using a Biorad iCycler iQ™ (Biorad, Hercules, CA, USA). The samples were then heated at 85 °C for 5 min and cooled down to 4 °C. The cDNA was stored at −80 °C for further use.

For complete cDNA amplification, we used 10× RT random primers in the reverse transcription kit and prepared the samples according to the manufacturer’s protocol. The cDNA was amplified with an initial step of 10 min at 25 °C, followed by 120 min at 60 °C, and a final heating step of 5 min at 85 °C.

### 2.7. Quantitative Polymerase Chain Reaction (qPCR)

qPCR was performed with Powerup SYBR Green Master Mix (A25776, Thermo Fisher, Applied Biosystems, Waltham, MA, USA) on a StepOnePlus real-time PCR system (Applied Biosystems) according to the manufacturer’s protocol. We used self-designed primers and pre-designed primers found in the literature (Tables S2–S4). The cycling conditions for the miRNA qPCR included initial holding stages for 2 min at 50 °C and 2 min at 95 °C, followed by 50 cycles of 15 s at 95 °C and 30 s at 60 °C. For complete cDNA, the conditions were 2 min at 50 °C, 10 min at 95 °C, followed by 60 cycles of 15 s at 95 °C and 1 min at 60 °C. For evaluation, we used the 2^−ΔΔCt^ method and normalized our miRNA expression to RNU-6 and mRNA expression to GAPDH.

### 2.8. Cell Proliferation

Cell growth and proliferation were assessed by seeding 1000 cells per well of control and HGPS fibroblasts in 24-well plates and transfecting after 24 h with a mimic/inhibitor and their respective negative control (see Section 2.3). Cells were counted every second day using a Muse™ Count and Viability reagent (MCH100102, Cytek Biosciences, Fremont, CA, USA) on a Muse cell analyzer (Merck Millipore, Burlington, MA, USA).

### 2.9. Western Blot

Cultured cells were harvested using a self-prepared lysis buffer consisting of 150 mM Sodium Chloride (S7653, Sigma-Aldrich), 50 mM Tris Base (T1503, Sigma Aldrich), 1 mM Ethylenediaminetetraacetic acid (EDTA, E9884, Sigma-Aldrich), 1% Triton X-100 (T-8787, Sigma-Aldrich), and 1% Sodium Dodecyl Sulfate (SDS, L3771, Sigma-Aldrich) adjusted to pH 8.0. The lysis buffer was mixed in a 1:1 ratio with 2× Laemmli buffer (1610737, Biorad) with the addition of Halt™ Protease and Phosphatase inhibitor (Thermofisher, 78440), β-Mercaptoethanol (1610710, Biorad), and 200 mM Phenylmethylsulfonyl fluoride (PMSF; 8553S, Cell Signaling). Cells were scraped from the culture dish, vortexed, and incubated at 95 °C for 5 min. Concentrations were determined using Bradford assay kit (5000202EDU, Biorad). We loaded 10 µg of cell lysate onto an 8% gel and conducted electrophoresis at 100 V for about 2.5 h. Proteins were transferred onto nitrocellulose membranes (GE10600003, Sigma-Aldrich) for 1.5 h at 400 mA. Membranes were blocked in 5% non-fat milk and incubated with primary antibodies: SIRT1 (D1D7) rabbit mAb (#9475, Cell Signaling, 1:1000), p21 Waf1/Cip1 (DCS60) mouse mAb (#2946, Cell Signaling, 1:2000), and GAPDH rabbit mAb (G9545, Sigma-Aldrich, 1:5000). After incubation of the primary antibody overnight at 4 °C (for GAPDH: 1 h at room temperature) blots were washed in Tris-buffered saline (TBS)–Tween for 3 × 5 min and incubated with peroxidase AffiniPure Goat anti-rabbit IgG (111035003, Jackson Immuno Research, West Grove, PA, USA) and peroxidase AffiniPure Goat anti-mouse IgG (115035003, Jackson Immuno Research) for 1 h at room temperature. Following a final wash (3 × 5 min), signals were displayed using Clarity Western ECL Substrate (1705061, Bio-Rad, Hercules, CA, USA) on a ChemiDoc™ MP visualizer (Bio-Rad) and quantified with the ImageLab Software (Version 6.1, Bio-Rad) or Image Studio Lite Version 5.2 (Li-Cor). Protein signals were normalized using Glycerin-aldehyde-3-phosphate dehydrogenase (GAPDH) as the internal control.

### 2.10. Immunofluorescence

Cells were grown on small cover glasses (631-1577P, VWR International GmbH, Darmstadt, Germany) and fixed with 2% Paraformaldehyde (1004005, Merck) in PBS for 10 min. After two steps of washing with PBS (D8537, Sigma-Aldrich), the cells were permeabilized with 0.1% Triton X-100 (T8787, Sigma) in PBS for 5 min and then washed again once with PBS. The cells were then blocked using 15% fetal bovine serum (10270106, Thermofisher, Gibco) in PBS for 30 min. After blocking, the coverslips were stained overnight with anti-Actin, alpha-smooth muscle-Cy3™ (mouse, 1:500, Sigma, C6198). After three washing steps with the blocking buffer and two with PBS, the cells were counterstained with DAPI mounting medium (Vectashield, Biozol, H-1200). Pictures were taken using a BZ-X810 fluorescence microscope (Keyence, Osaka, Japan).

### 2.11. Mouse Skin qPCR Experiments

We used skin samples from the knock-in mouse model C57BL6 LMNA^G609G/G609G^ described by Osorio et al. [44,45]. All animal procedures were approved by the Animal Care and Ethics Committee of the Institutional Review Board of the Government of Upper Bavaria (reference: ROB-55.2-2532. Vet_02-19-72, 12 September 2019). At the age of 90 days, three male homozygous and age-matched wildtype mice were euthanized, and skin samples were taken from the back after removal of the hair. The skin samples were frozen in liquid nitrogen and stored at −80 °C. For RNA isolation, small pieces of the skin were cut and disrupted using a mortar and pestle and under liquid-nitrogen cooling. For lysis, the skin pieces were dissolved in QIAzol Lysis Reagent (79306, Qiagen) with a Dounce tissue homogenizer (CXE1.1, Carl Roth, Karlsruhe, Germany). The RNA was extracted with the miRNeasy Mini Kit (217004, Qiagen) according to the manufacturer’s protocol. miRNAs and target gene expression were quantified by reverse transcription and qPCR, as described above.

### 2.12. Statistics

Statistical analysis of the conditions, comparing the control and HGPS fibroblasts in different growth stages and treatment conditions, was performed using two-way ANOVA with Tukey’s post hoc test. Comparisons between control and HGPS mouse tissues were statistically analyzed with an unpaired *t*-test. *p* < 0.05 was assumed to be statistically significant. All statistical analyses were calculated in GraphPad Prism 8.0.

## 3. Results

### 3.1. Text-Mining Approach to Determine miRNAs Associated with Autophagy and Inflammation

**Figure 1:**
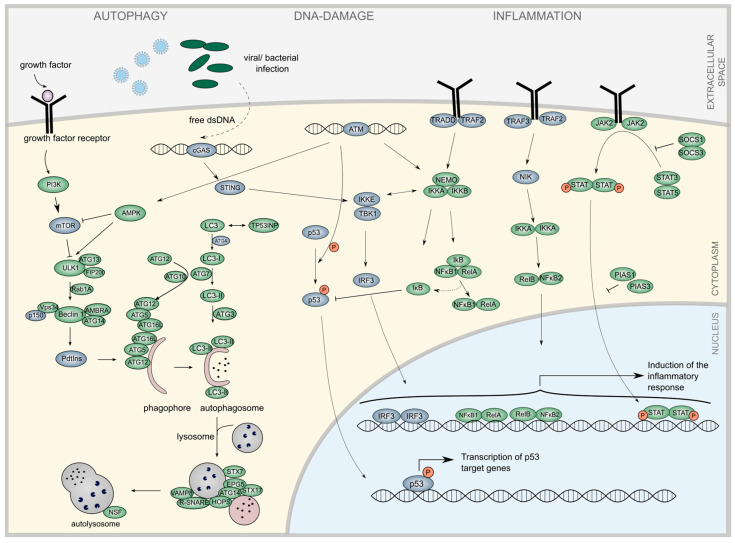
Interconnection between autophagy and inflammatory pathways. Under high-nutrient conditions, the mechanistic target of rapamycin (mTOR) is activated, leading to the inhibition of the autophagic pathway, which involves the initiation, elongation, maturation, and fusion of the autophagosome [37,46,47]. Inflammation signaling typically results in the activation of NF-κB, which can occur through canonical or non-canonical mechanisms. The canonical activation of NF-κB is initiated by the binding of growth factors to tumor necrosis factor (TNF) or toll-like receptors (TLRs), which activate TNF receptor-associated factor (TRAF) proteins. This leads to the activation of the IKK complex, degradation of IKB, and translocation of the NF-ĸB1-RelA complex into the nucleus to activate proinflammatory genes. In contrast, the non-canonical pathway is triggered by signals from TNF receptors that activate the NF-ĸB-inducing kinase, leading to IKK activation [48,49,50,51]. TLR4 signaling also induces the JAK/STAT pathway, another critical component of inflammatory signaling [24,52,53]. Autophagy and inflammation are connected through the DNA damage response. DNA damage activates the cytosolic DNA sensors cGAS and STING, which, in conjunction with ATM, can stimulate both inflammatory responses and autophagic vesicle formation, as well as the central cell-cycle regulator p53 [54,55,56,57]. Proteins analyzed by miRNA interactions are illustrated in green.

We performed a comprehensive literature search to identify miRNAs linked to two hallmarks of HGPS: autophagy and inflammation (Figure 1). Autophagy is a critical cellular function involved in the degradation of misfolded proteins and is essential for maintaining cellular homeostasis. Regulated by the mechanistic target of rapamycin (mTOR), autophagy involves a complex maturation process that results in lysosomal fusion and degradation of target proteins [37,46,47] (Figure 1). Closely linked to the degradation of harmful or redundant proteins is inflammatory signaling, which mostly results in the canonical or non-canonical activation of NF-ĸB [48,49,50,51]. TLR4 signaling constitutes another branch of the inflammatory network, and leads to JAK1/2-STAT1/3 signaling [24,52,53]. A connection between autophagic and inflammatory pathways is the DNA damage response, which connects the recognition of foreign or damaged DNA to intracellular signaling. Through activation of p53, central cell-cycle regulation processes are induced, linking DNA damage responses to both inflammation and autophagy, thereby providing an efficient defense against pathogens and other DNA damage inducers (Figure 1) [54,55,56,57].

**Figure 2:**
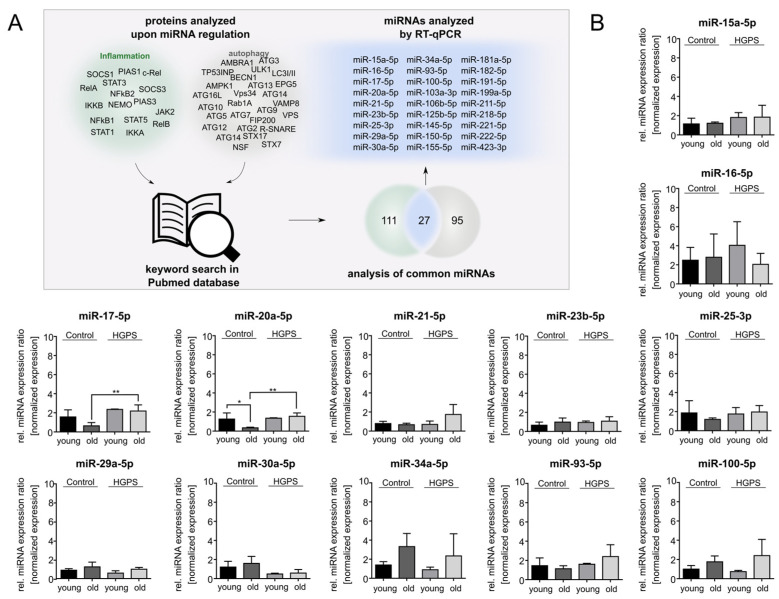

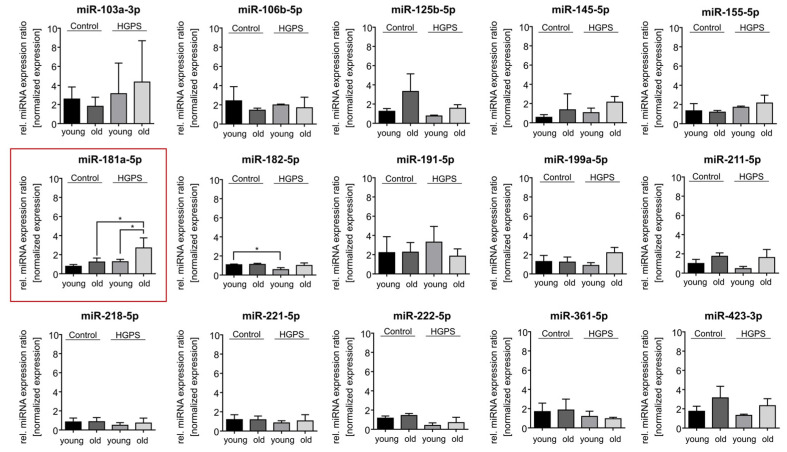
Identification and profiling of miRNAs regulating autophagy and inflammation pathways in control and HGPS. (**A**) Literature search procedure. Proteins from both autophagy and inflammation pathways were examined for miRNA regulation, and overlapping miRNAs were analyzed using RT-qPCR. (**B**) RT-qPCR analysis. miRNAs identified through text mining were analyzed in normal and HGPS fibroblasts during replicative senescence. Control fibroblasts (GMO1651c, GMO1652c, GMO3349c, GMO1582B) and HGPS fibroblasts (HGADFN127, HGADFN188, HGADFN003) were compared at passages with a replicative senescence under 5% (young) and over 20% (old) intragroup, as well as same-aged groups. Relative expression levels were normalized to U6 small nuclear 1 (RNU-6). Graphs show mean values ± standard deviation. Statistical significance was calculated using ANOVA with Tukey’s post hoc test (* *p* < 0.05, ** *p* < 0.01, (control: *n* = 4, HGPS: *n* = 3)). Non-significant changes are not indicated.

In progeroid cells, progerin attaches to the nuclear envelope, deforms the nucleus, and inhibits its function [19,58]. However, defective autophagic degradation of progerin further exacerbates inflammatory responses, contributing to HGPS progression [14,19,24,59,60]. Central mediators of accelerated inflammatory responses, such as IL-6 signaling and both canonical and non-canonical NF-ĸB signaling, have been shown to be deregulated in HGPS (Figure 1) [25,26,61,62]. To elucidate the regulatory role of miRNAs in these interconnected processes, we analyzed the pathways involved in HGPS pathogenesis and assessed how miRNA dysregulation may contribute to disease progression [13,24]. Given the potential interplay between autophagy and inflammation in HGPS, we investigated miRNAs that modulate these pathways. Through a systematic text-mining approach evaluating the interacting miRNAs of key autophagy and inflammatory proteins, we identified 138 miRNAs associated with inflammation and 122 miRNAs linked to autophagy-related proteins. We then cross-referenced these datasets to pinpoint miRNAs that regulate both pathways (Figure 2A). Candidate miRNAs with dual regulatory roles were subsequently validated using RT-qPCR.

### 3.2. miR-181a-5p Is Upregulated in HGPS Fibroblasts During Replicative Senescence

To investigate the impact of miRNAs on HGPS progression, we cultured HGPS primary fibroblasts (HGADFN127, HGADFN003, HGADFN188) alongside control fibroblasts (GMO1651c, GMO1652c, GMO3349c, GMO1582B) and performed qPCR screenings for the 27 overlapping miRNAs (Figure 2B). To differentiate between early- and late-passage fibroblast cultures, we determined the senescence index. Young fibroblasts exhibit a low senescence index (<5%) and remain highly proliferative, whereas old cultures with a senescence index over 20% show reduced replicative potential [63,64]. The senescence index thus provides a passage-independent measurement of cellular aging by detecting senescence-associated β-Galactosidase activity, a hallmark of senescent cells [41]. This method enabled normalization of the relative age of fibroblast cultures for miRNA expression analysis. qPCR analysis revealed distinct miRNA (miR) expression patterns during cellular aging. miR-20a-5p was downregulated in aging of control fibroblasts, whereas this decline was not observed during aging of HGPS fibroblasts. Comparing old control and old HGPS cells, miR-17-5p and miR-20-5p were significantly increased in HGPS cells (Figure 2B). miR-181a-5p was markedly upregulated in old HGPS fibroblast cultures compared to their younger counterparts. Additionally, miR-182-5p was downregulated in young HGPS cells compared to the controls (Figure 2B). Other miRNAs displayed trends of up- or downregulation. However, these changes were not statistically significant (Figure 2B).

Notably, miR-181a-5p emerged as a key candidate, as it was the only miRNA specifically upregulated in HGPS fibroblasts during cellular aging (Figure 2B, red box). Since progerin accumulation has been consistently observed in HGPS during cellular aging in several studies, we propose a strong correlation between miR-181a-5p and the progerin-associated alterations in signaling pathways [65,66]. Based on this, we further investigated its regulatory function and downstream targets.

Using an integrated literature approach, we curated a preselected list of miR-181a-5p targets and interacting proteins, grouping them by functional categories (Table 2). This analysis not only validated key targets from our initial text-mining screen but also identified novel targets involved in cell-cycle regulation, energy metabolism, and DNA damage response (Table 2).

**Figure 3:**
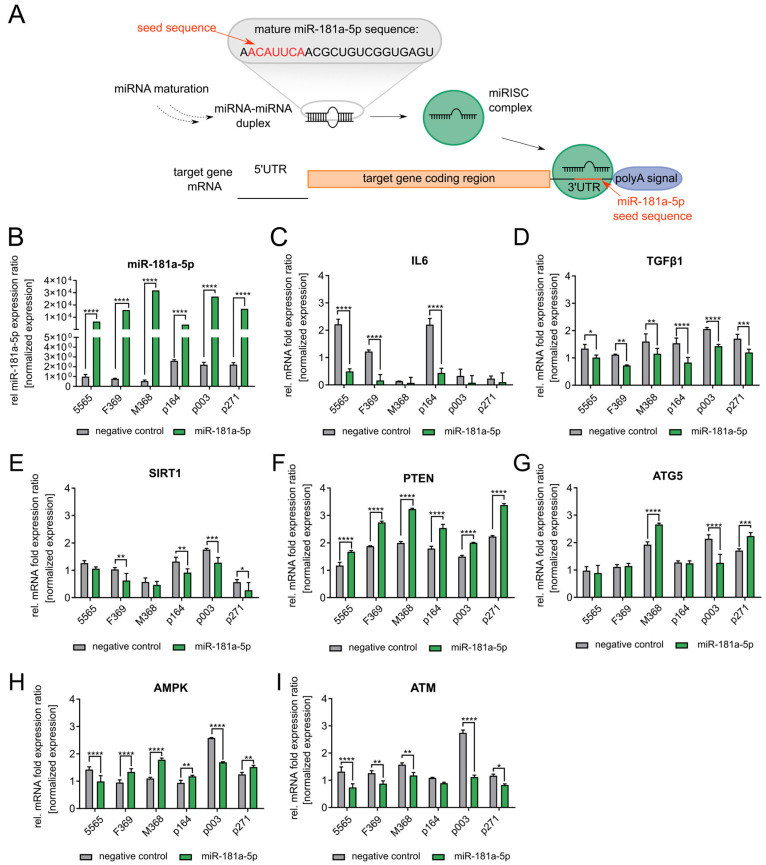
Evaluation of miR-181a-5p’s predicted targets using an miR-181a-5p mimic. Cell strains used: Control (GMO5565, HGFDFN369, HGMDFN368) and HGPS (HGADFN003, HGADFN164, HGADFN271) fibroblasts with a senescence index lower than 5%. (**A**) Schematic representation of miR-181a-5p’s binding to the target gene. After miRNA maturation, an miRNA-miRNA duplex is formed and loaded into the miRISC complex to recognize the seed sequence of the miRNA at the 3′UTR and repress protein translation of the target gene. (**B**) Relative miR-181a-5p expression upon miR-181a-5p mimic treatment for 6 days. (**C**) Relative mRNA expression levels of IL6. (**D**) Relative mRNA expression levels of TGFβ1. (**E**) Relative mRNA expression levels of SIRT1. (**F**) Relative mRNA expression levels of PTEN. (**G**) Relative expression levels of ATG5. (**H**) Relative mRNA expression levels of AMPK. (**I**) Relative mRNA expression levels of ATM. (**B**–**I**) Expression of protein levels was normalized to GAPDH, and expression of miRNA levels was normalized to RNU6. Graphs show mean values ± standard deviation. Statistical significance was calculated using ANOVA with Tukey’s post hoc test (* *p* < 0.05, ** *p* < 0.01, *** *p* < 0.001, **** *p* < 0.0001 (*n* = 3)). Comparisons were made between negative control-treated and miR-181a-5p mimic-treated samples in the same cell lines. Non-significant changes are not indicated.

The targets have miR-181a-5p’s seed sequence near the 3′ untranslated region (UTR) of their mRNAs, suggesting direct post-transcriptional regulation (Figure 3A). To validate these interactions, we transfected an miR-181a-5p mimic into three control and HGPS cell strains and observed a 5000 to 30,000-fold increase in miR-181a-5p levels depending on the cell strain (Figure 3B). The substantial elevation provides a model to assess its functional role in HGPS pathophysiology. To maintain cellular proliferation after transfection and obtain a complete view of the cellular processes induced by the miRNA, we used early-passage (young) fibroblast strains with a senescence rate below 5%. To account for heterogeneity between cell strains, we analyzed each fibroblast strain individually. To determine whether miR-181a-5p influences inflammatory pathways, we analyzed the senescence-associated secretory phenotype (SASP) markers IL-6 and TGFβ1 via RT-qPCR. IL-6 showed a decreased trend but this was not statistically significant across all cell strains, whereas TGFβ1 protein levels were significantly decreased following miRNA mimic treatment (Figure 3C,D). As IL-6 is suggested to be a direct target of miR-181a-5p, our findings suggest that miR-181a-5p contributes to an overall anti-inflammatory effect by downregulating IL-6 expression. The regulation of TGFβ1, an upstream inducer of miR-181a-5p, suggests a functional crosstalk in the modulation of miR-181a-5p expression under inflammatory conditions. The 6-day treatment period may have limited the full extent of miR-181a-5p-mediated inflammatory modulation, potentially due to compensatory interactions with additional regulatory factors. Moreover, the high inter-strain variability and differential responses to the miR-181a-5p mimic must be considered, which complicates drawing conclusions about the role of miR-181a-5p in modulating inflammatory pathways. Next, we investigated SIRT1 expression following miR-181a-5p mimic treatment. The qPCR results demonstrated a significant downregulation of SIRT1 mRNA in most transfected fibroblasts (Figure 3E). To further explore miR-181a-5p’s direct effects on cell survival, we analyzed the expression of PTEN in control and HGPS fibroblasts (Figure 3F). PTEN levels were significantly increased in all miR-181a-5p mimic-treated cells (Figure 3F). As PTEN is typically downregulated by miR-181a-5p via binding to its 3′UTR, our findings suggest a more complex regulatory interaction influencing PTEN expression (Figure 3F) [70]. Similarly, the autophagy-related proteins ATG5 and AMPK were expected to be downregulated by miR-181a-5p. However, during mimic treatment, we observed high variability in ATG5 and AMPK mRNA levels (Figure 3G,H). This supports a model in which miR-181a-5p exerts context-dependent regulatory effects on autophagy, strongly influenced by cellular heterogeneity among different fibroblast strains (Figure 3G,H). To further evaluate the targets and influence of miR-181a-5p, we measured the mRNA expression of the DNA damage response regulator ATM (Figure 3I). During miR-181a-5p mimic treatment, ATM expression was downregulated, in most strains significantly. These results suggest that miR-181a-5p may influence the DNA damage response; however, this potential role requires further investigation (Figure 3I).

**Figure 4:**
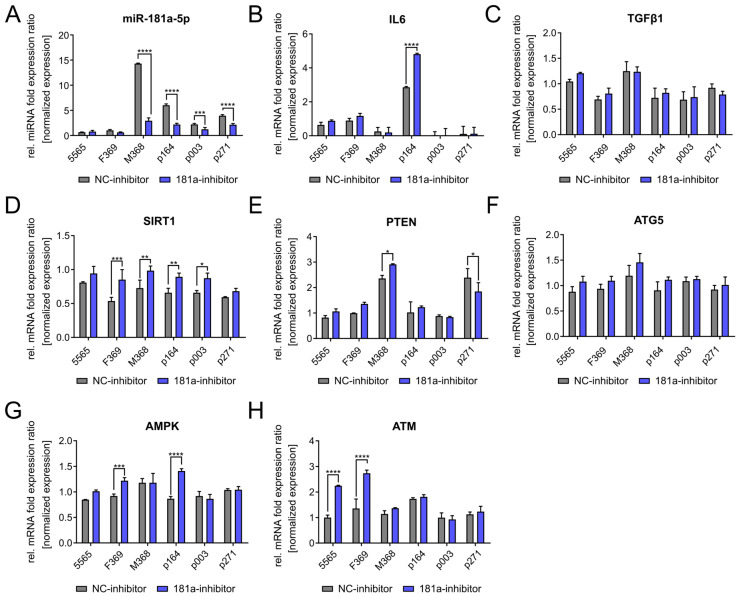
Evaluation of miR-181a-5p’s predicted targets using an miR-181a-5p inhibitor. Cell strains used: Control (GMO5565, HGFDFN369, HGMDFN368) and HGPS (HGADFN003, HGADFN164, HGADFN271) fibroblasts with a senescence index lower than 5%. (**A**) Relative miR-181a-5p expression upon miR-181a-5p inhibitor treatment for 6 days. (**B**) Relative mRNA expression levels of IL6. (**C**) Relative mRNA expression levels of TGFβ1. (**D**) Relative mRNA expression levels of SIRT1. (**E**) Relative mRNA expression levels of PTEN. (**F**) Relative mRNA expression levels of ATG5. (**G**)—Relative mRNA expression levels of AMPK. (**H**) Relative mRNA expression levels of ATM. (**A**–**H**) Expression of protein levels was normalized to GAPDH, and expression of miRNA levels was normalized to RNU6. Graphs show mean values ± standard deviation. Statistical significance was calculated using ANOVA with Tukey’s post hoc test (* *p* < 0.05, ** *p* < 0.01, *** *p* < 0.001, **** *p* < 0.0001 (*n* = 3)). Comparisons were made between negative control-treated and miR-181a-5p mimic-treated samples in the same cell lines. Non-significant changes are not indicated.

To further confirm our results, we transfected an miR-181a-5p inhibitor into the same cell strains and analyzed the mRNA expression levels of the potential target genes. As shown in Figure 4A, miR-181a-5p levels were significantly reduced in most cell strains following inhibitor treatment. Also, miR-181a-5p levels varied among negative control-treated cells, likely due to a transfection-induced stress response [73]. When comparing the IL-6 mRNA expression following inhibitor treatment, we observed only a partial effect (Figure 4B). TGFβ1 expression remained unchanged during this treatment (Figure 4C). In contrast, significant changes in SIRT1 mRNA levels were observed in most cell strains, indicating the critical role of miR-181a-5p in SIRT1 regulation (Figure 4D). PTEN, AMPK, ATG5, and ATM mRNA expression levels were not significantly changed and showed no consistent trend of increase or decrease (Figure 4E–H).

The target validation experiments using miR-181a-5p mimic and inhibitor treatments suggest an overall anti-inflammatory effect of miR-181a-5p during the 6-day treatment period, as shown by the downregulation of IL-6 and TGFβ1, as well as significant downregulation of the DNA damage response regulator ATM during mimic treatment. These effects could not be reversed by inhibitor treatment, suggesting a more complex regulatory mechanism or a distinction between excessive miR-181a-5p mimic transfection and physiological-level inhibition. The impact on mRNA expression is pronounced when miR-181a-5p is administrated beyond physiological levels and differs from the effect observed in inhibitor experiments. ATG5 and AMPK were not changed under either mimic or inhibitor treatment, indicating a limited role of miR-181a-5p in autophagic regulation. PTEN mRNA expression was significantly increased during miR-181a-5p mimic treatment, but this effect was not consistently reversed by inhibitor treatment across all cell strains. Additionally, high variability in mRNA expression across different cell strains makes it difficult to draw conclusions about regulatory mechanisms. Since PTEN is suggested to be downregulated by miR-181a-5p via binding to its 3′UTR, our findings suggest a more complex regulatory mechanism may enhance PTEN expression. SIRT1 emerged as the most consistently downregulated transcript during miR-181a-5p mimic treatment, with a corresponding increase observed following miR-181a-5p inhibitor treatment. This significant upregulation under physiological inhibition, along with significant PTEN increase during miR-181a-5p mimic treatment, led us to examine p53, a central cell-cycle regulator that is negatively regulated by SIRT1-mediated deacetylation. p53 is known to transcriptionally upregulate PTEN, which may explain the increased PTEN mRNA levels observed following miR-181a-5p overexpression [74,75]. A major downstream target of p53 is p21 (CDKN1A), a cyclin-dependent kinase inhibitor that induces G1-phase arrest and promotes senescence [76,77,78]. p21 is elevated during aging and is increased earlier in HGPS cells [78].

### 3.3. miR-181a-5p Inhibits SIRT1 and Regulates Cell Growth and Senescence

**Figure 5:**
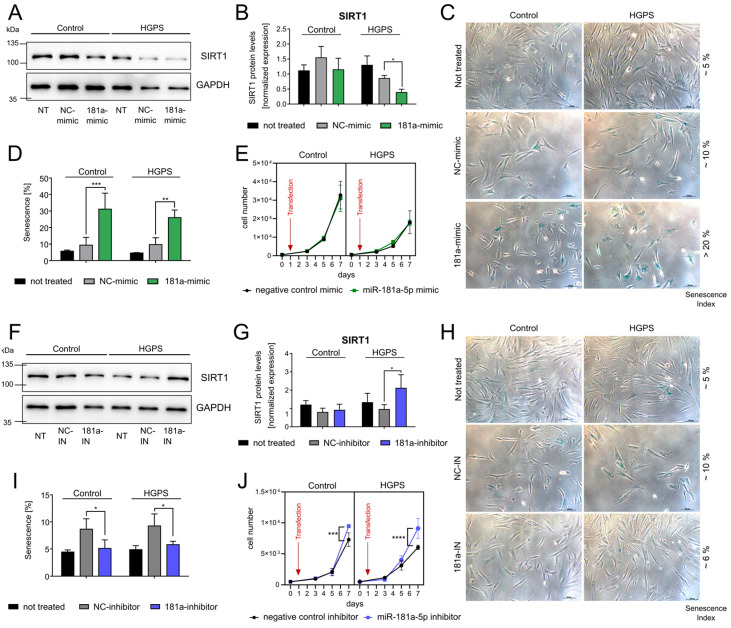
Impact of miR-181a-5p regulation on cell growth and senescence. Fibroblast strains used: Control—GMO5565, HGFDFN369, HGMDFN368; HGPS—HGADFN003, HGADFN271, HGADFN164. Abbreviations: NT—non-treated, NC mimic—negative control mimic, 181a mimic—miR-181a-5p mimic, NC-IN—negative control inhibitor, 181a-IN—miR-181a-5p inhibitor. (**A**) Representative Western blot analysis of SIRT1 and GAPDH as loading control during miR-181a-5p mimic treatment in control (HGMDFN368) and HGPS (HGADFN271) fibroblasts with a senescence index <5%. (**B**) Quantification of SIRT1 protein levels normalized to GAPDH. (**C**) Representative brightfield images of control (GMO5565) and HGPS (HGADFN164) fibroblasts stained with ß-Galactosidase assay during miRNA mimic treatment for 9 days. Scale bar = 100 µm. (**D**) Replicative senescence determined by ß-Galactosidase assay after 9-day miR-181a-5p mimic treatment. (**E**) Representative growth curve of young control (GMO5565) and HGPS (HGADFN003) cell strains during miR-181a-5p mimic treatment for 6 days. (**F**) Representative Western blot analysis of SIRT1 and GAPDH as loading control during miR-181a-5p inhibitor treatment in control (HGMDFN368) and HGPS (HGADFN271) fibroblasts with a senescence index <5%. (**G**) Quantification of SIRT1 protein levels normalized to GAPDH. (**H**) Brightfield images of control (GMO5565) and HGPS (HGADFN164) fibroblasts stained with ß-Galactosidase assay during miRNA inhibitor treatment for 9 days. Scale bar = 100 µm. (**I**) Replicative senescence determined by ß-Galactosidase assay after 9-day miR-181a-5p inhibitor treatment. (**J**) Representative growth curve of young control (GMO5565) and HGPS (HGADFN003) cell strains during miR-181a-5p inhibitor treatment for 6 days. (**A**–**J**) Graphs show mean values ± standard deviation. Statistical significance was calculated using ANOVA with Tukey’s post hoc test (* *p* < 0.05, ** *p* < 0.01, *** *p* < 0.001 (*n* = 3)). Comparisons were made between negative control-treated and miR-181a-5p mimic-treated samples. Non-significant changes are not indicated.

To test whether miR-181a-5p regulates senescence and cell-cycle progression, we transfected three control and HGPS fibroblast strains with an miR-181a-5p mimic. Following transfection, miR-181a-5p levels increased by 5000- to 30,000-fold depending on the strains (Figure 3B). As shown in Figure 5A,B, SIRT1 protein levels were reduced in both control and HGPS fibroblasts after miR-181a-5p mimic transfection (Figure 5A,B; corresponding full-scan blots, Figure S2A,B). However, the downregulation in control cells was not statistically significant. In contrast, p21 (CDKN1A), a key downstream target of p53, was significantly upregulated in control fibroblasts, although it did not reach statistical significance in HGPS cells (Figure S1A,B; corresponding full-scan blots, Figure S3). To assess the impact of miR-181a-5p on senescence, we treated fibroblasts with the miR-181a-5p mimic for 9 days and monitored senescence progression. As shown in Figure 5C,D, senescence levels increased following negative control transfection and were further elevated upon miR-181a-5p overexpression. The increase in senescence during negative-control transfection likely reflects transfection-induced cellular stress, which is known to activate proinflammatory cytokines and SASP markers [73]. Despite no changes in fibroblast growth after 6 days of miR-181a-5p mimic treatment, we anticipate a progressive decline in growth rate upon prolonged exposure due to senescence induction (Figure 5E). Given that miR-181a-5p is downregulated in various cancers and implicated in tumor suppression, we hypothesized that inhibiting miR-181a-5p would delay senescence [79,80]. To test this, we transfected control and HGPS fibroblasts with the miR-181a-5p inhibitor. As shown in Figure 4A, miR-181a-5p levels were significantly reduced in most cell strains following inhibitor treatment. Western blot analysis of SIRT1 after miR-181a-5p inhibitor treatment showed a restoration of SIRT1 protein levels (Figure 5F,G and Figure S2C,D). By day 9 of miR-181a-5p-inhibitor treatment, we observed a significant reduction in the proportion of senescent cells (Figure 5H,I). The increase in senescence with the negative control inhibitor treatment is again likely due to transfection-induced stress [73,81]. Fibroblast proliferation was significantly enhanced after 6 days of inhibitor treatment in both control and HGPS cells, confirming the restoration of cellular proliferation and cellular homeostasis by miR-181a-5p inhibition (Figure 5J). As expected, we also observed a decreasing trend of p21 protein levels. Although these changes were not significant after 6 days of treatment, the trend supports the attenuation of senescence observed after 9 days (Figure S1C,D; corresponding full-scan blots, Figure S3).

### 3.4. TGFβ1 Upregulates miR-181a-5p Expression in Normal and HGPS Fibroblasts

Upon examining the regulatory mechanisms of miR-181a-5p, we identified TGFβ1 as a potential activator, suggesting that miR-181a-5p upregulation may be driven by an enhanced inflammatory response [68,82].

**Figure 6:**
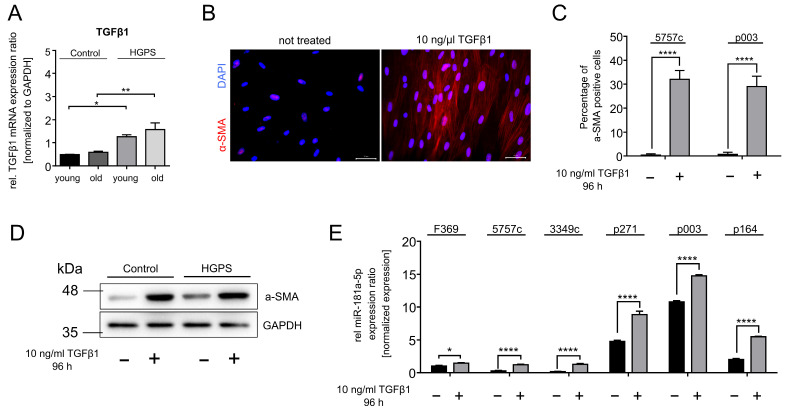
Elevated TGFβ1 levels in HGPS promote upregulation of miR-181a-5p expression. (**A**) TGFβ1 mRNA expression level in three control (GMO5565, GMO5757c, HGFDFN369) and HGPS (HGADFN127, HGADFN003, HGADFN164) fibroblast cell strains. Comparisons were made between the young and old stages intragroup as well as same-aged stages. Expression was normalized to GAPDH. (**B**) Representative immunofluorescence staining of the control cell line 5757c P18 with α-SMA following TGFβ1 treatment. Scale bar = 50 µm (**C**) Quantification of immunofluorescence signal in control (GMO5757c P18) and HGPS (HGADFN003 P12) cell strains. Comparisons were made between treated and non-treated samples. (**D**) Western blot of α-SMA and GAPDH as a loading control in control (GMO5757c P18) and HGPS (HGADN271 P11) cell strains. (**E**) Relative miR-181a-5p levels following TGFß1 treatment in three control (HGFDFN369, GMO5757c, GMO3349c) and HGPS (HGADFN271, HGADFN003, HGADFN164) cell strains with a senescence index <5%. Expression was normalized to U6 small nuclear 1 (RNU-6). Comparisons were made between treated and non-treated samples. (**A**,**C**,**E**) Graphs show mean values ± standard deviation. Statistical significance was calculated using ANOVA with Tukey’s post hoc test (* *p* < 0.05, ** *p* < 0.01, **** *p* < 0.0001 (*n* = 3)).

TGFβ is a key regulator of inflammation, modulating a broad spectrum of miRNAs [82,83]. In HGPS, TGFβ1 mRNA expression is significantly upregulated when comparing young controls with young HGPS cell strains, as well as their aged counterparts (Figure 6A). To investigate whether TGFβ1 causes an induction of miR-181a-5p, we treated young HGPS and control fibroblasts with 10 ng/ml TGFβ1 for four days and measured miR-181a-5p expression. To confirm cellular uptake and downstream signaling, we performed immunofluorescence staining and Western blot analysis for α-smooth muscle actin (α-SMA), a known marker of TGFβ1-induced myofibroblast differentiation [84]. As shown in Figure 6B, α-SMA expression was induced in TGFβ1-treated control fibroblasts, as indicated by the enhanced cytoskeletal signal. Immunofluorescence staining further demonstrated a significant increase (~30%) in α-SMA expression in the treated groups, confirming effective TGFβ1 induction (Figure 6C). Western blot analysis corroborates these findings, showing a clear increase of α-SMA in TGFβ1-treated fibroblasts (Figure 6D). Next, we analyzed miR-181a-5p levels in TGFβ1-treated fibroblasts and observed a significant upregulation, with increases ranging from 1.5-fold to 7-fold, depending on the cell strain (Figure 6E). These findings confirm that TGFβ1 is a potent inducer of miR-181a-5p expression in both normal and HGPS fibroblasts, reinforcing its role as an upstream regulator in inflammatory signaling.

### 3.5. miR-181a-5p Expression Levels Are Deregulated in a Mouse Model of HGPS

**Figure 7:**
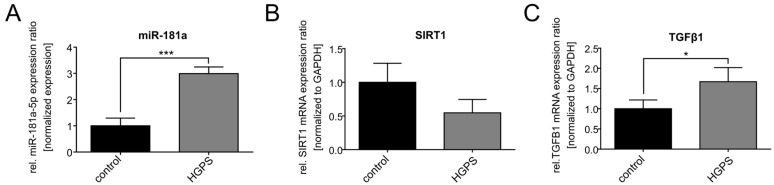
In vivo validation of the miR-181a-5p interaction with SIRT1 and TGFβ1. (**A**) miR-181a-5p levels in wildtype versus homozygous mouse skin samples. Signals were normalized to RNU-6. (**B**) Relative SIRT1 mRNA expression levels in wildtype versus homozygous mouse skin samples. Signals were normalized to GAPDH. (**C**) Relative TGFβ1 mRNA expression levels in wildtype versus homozygous mouse skin samples. Signals were normalized to GAPDH. (**A**–**C**) Graphs show mean values ± standard deviation. Statistical significance was calculated using an unpaired *t*-test (* *p* < 0.05, *** *p* < 0.001, (*n* = 3)). Non-significant changes are not indicated.

Building on our findings from the fibroblast studies, we extended our investigation to an in vivo model to assess miR-181a-5p expression in skin biopsies from an HGPS mouse model. We analyzed the Lmna^G609G/G609G^ mouse model and wildtype counterparts [44]. In 90-day-old male mice, we observed a three-fold upregulation of miR-181a-5p in skin samples from homozygous mice compared to wildtype controls (Figure 7A). SIRT1 mRNA levels showed a trend toward downregulation in homozygous mice relative to wildtype, but the difference was not statistically significant (Figure 7B). In contrast, the mRNA levels of TGFβ1, a known inducer of miR-181a-5p, were significantly increased in the skin of Lmna^G609G/G609G^ mice (Figure 7C). These results suggest a critical role of miR-181a-5p in vivo and highlight its physiological relevance in HGPS disease progression.

## 4. Discussion

A growing body of research is focused on understanding the progression of the HGPS phenotype and identifying strategies to counteract progerin-induced cellular dysfunction. Despite significant advancements, there is currently no curative treatment for HGPS. The only FDA-approved drug for HGPS, lonafarnib, a farnesyltransferase inhibitor, partially reduces nuclear blebbing and restores nuclear membrane integrity [7,85]. Clinical studies have shown that lonafarnib improves overall health and extends the median lifespan by approximately 1.6 years in HGPS patients [86,87]. Beyond farnesyltransferase inhibitors, several other treatment strategies have been investigated to limit the detrimental effects of progerin. For example, ICMT inhibitors aim to reduce progerin mislocalization by inhibiting the carboxyl methylation of farnesylated progerin, thereby improving cell viability and ameliorating key HGPS hallmarks [88]. Compounds that block the interaction between progerin and lamin A have gained increasing interest, as they help restore nuclear morphology and reduce cellular senescence [89]. To eliminate progerin accumulation, gene therapy approaches such CRISPR/Cas9 genome editing aim to correct the *LMNA* mutation [90]. Additionally, therapeutic strategies have been developed to target HGPS at both the RNA and DNA levels. Antisense oligonucleotides (ASOs) have been developed to shift aberrant LMNA splicing toward Lamin C production, demonstrating the potential of RNA-based interventions [44,91]. In addition, non-coding RNAs, such as miRNAs, have gained attention for their regulatory roles in HGPS pathogenesis. For example, the brain-specific miRNA miR-9 downregulates Lamin A and progerin expression in neural tissues, thereby protecting neurons from premature aging phenotypes [31]. This finding shows the need for further investigation into the role of miRNAs in HGPS disease progression. Our study focused on two major dysregulated pathways in progeria: autophagy and inflammation. Given the significant impact of progerin on the pathways, we hypothesized that specific miRNAs regulating both processes would also be altered in HGPS. Through extensive literature screening, we identified 27 overlapping miRNAs and analyzed their expression profiles in young and aged HGPS and control fibroblasts using stem-loop RT-qPCR. Among these, miR-181a-5p emerged as the most prominent candidate, exhibiting significant upregulation in aging HGPS fibroblasts, leading us to select it for further investigation. miR-181a-5p is a well-investigated miRNA implicated in various cancer types [79]. However, its function varies across different cell types. In glioma, miR-181a-5p upregulation promotes G1-phase arrest by targeting caspase-9, Bcl-2, and SIRT1, ultimately inhibiting cell proliferation [80]. In acute myeloid leukemia, miR-181a-5p and its enhancer TGFβ are both downregulated [92]. Our findings align with these studies, as miR-181a-5p was upregulated in HGPS fibroblasts and late-passage (old) cultures, further supporting its potential as a therapeutic target in HGPS. Building on our findings, we identified TGFβ1 as a key upstream inducer of miR-181a-5p through computational prediction and experimental validation [82]. We confirmed this in control and HGPS fibroblasts, where we observed a significant increase in miR-181a-5p expression (1.5- to 7-fold) following TGFβ1 treatment.

Analyzing the downstream effects of miR-181a-5p, we measured the mRNA expression of key targets. We observed downregulation of IL-6, a major mediator of the senescence-associated secretory phenotype (SASP) and systemic inflammation [93]. Previously, miR-181a-5p was shown to inhibit proinflammatory cytokines such as IL-6, IL-1a, and TNFα via TLR4 signaling [67]. Our findings are consistent with the anti-inflammatory role of miR-181a-5p. During miR-181a-5p inhibitor transfection, these interactions could not be validated, suggesting a more complex regulatory network. Variability in response across cell strains further limits interpretation, making it difficult to draw definitive conclusions regarding its role in inflammation. Most prominent during miR-181a-5p mimic treatment is the downregulation of SIRT1 mRNA levels and significant induction of PTEN, suggesting activation of the SIRT1-p53-p21 signaling axis. miR-181a-5p inhibitor treatment reversed the effect on SIRT1, resulting in increased mRNA expression. The DNA damage regulator ATM was significantly downregulated during miR-181a-5p mimic treatment; however, this effect could not be reversed during miR-181a-5p inhibitor treatment. Other targets, such as the autophagy-related proteins ATG5 and AMPK, did not show consistent changes in response to either miR-181a-5p mimic or inhibitor treatment, suggesting a limited role for miR-181a-5p in autophagy regulation. Importantly, cell strain-specific differences in response to miR-181a-5p mimic and inhibitor must be taken into account, as they contribute to variability in gene expression outcomes. These observations underscore the need for further investigation to understand the regulatory functions of miR-181a-5p in autophagy and inflammation

Nevertheless, further analysis of the SIRT1-p53-p21 regulatory axis revealed that SIRT1 protein expression was significantly downregulated following miR-181a-5p mimic treatment. Since SIRT1 contains an miR-181a-5p binding site in its 3′UTR, and previous studies have confirmed their direct interaction, our findings support an inverse regulatory relationship between miR-181a-5p and SIRT1 in both HGPS and aging [69]. This hypothesis is supported by reports indicating that SIRT1 is typically downregulated with age and in HGPS [94]. Downregulation of SIRT1 by miR-181a-5p leads to p53 activation, which in turn upregulates p21, a key effector of senescence [74]. We validated this by treating HGPS and control fibroblasts with an miR-181a-5p mimic for 9 days, followed by β-Galactosidase staining, which confirmed a significant increase in senescent cells. Conversely, miR-181a-5p inhibition resulted in enhanced cell proliferation, partial restoration of SIRT1 protein levels, and reduced senescence. Our study used multiple validation approaches, including miR-181a-5p mimic and antagomiR experiments, qPCR, and Western blot analyses, to assess downstream effects on target proteins such as SIRT1 and TGFβ. While mimic transfection resulted in miR-181a-5p levels exceeding physiological concentrations, our results indicate that miR-181a-5p is induced by TGFβ and interacts with SIRT1 under physiological conditions, supporting its potential role in HGPS pathogenesis.

To further validate our in vitro findings, we also analyzed miR-181a-5p expression in skin samples from Lmna^G609G/G609G^ mice, a well-established progeria model. qPCR confirmed a significant upregulation of miR-181a-5p in homozygous mice, along with a declining trend in SIRT1 mRNA levels. Previous studies have shown that inflammatory markers such as IL-6 are consistently elevated in HGPS mouse models, supporting a systemic inflammatory response during HGPS disease progression [95]. Additionally, TGFβ1 expression was significantly increased, further reinforcing its role in miR-181a-5p induction.

## 5. Conclusions

The upregulation of miR-181a-5p appears to be a response to chronic inflammation in HGPS, ultimately leading to SIRT1 suppression and cell-cycle arrest via p21 activation. Despite the significant changes in miR-181a-5p, SIRT1, and TGFβ1 in HGPS models and their known roles in senescence and HGPS progression, the limited number of biological replicates constrains the strength of our conclusions. Future studies with larger sample sizes will be necessary to validate the upregulation of miR-181a-5p in HGPS and further elucidate its impact on aging and disease pathogenesis. To strengthen the findings, monitoring of miR-181a-5p expression in response to progerin induction may also provide insights into their functional interplay. Future studies should further investigate the role of miR-181a-5p in HGPS progression and evaluate its potential as a therapeutic target in HGPS mouse models. Targeted modulation of miR-181a-5p expression may offer a promising therapeutic strategy to restore SIRT1 levels, counteract premature senescence in HGPS, and potentially influence normal aging processes.

## Figures and Tables

**Table 1:** Passage numbers relative to the senescence index of each fibroblast strain.

Cell Strain	Abbreviation	Young Culture (Senescence Index < 5%)	Old Culture (Senescence Index > 20%)
GMO1582B	1582B	Passage 10–14	Passage 18–20
GMO1651c	1651c	Passage 15–18	Passage 23–25
GMO1652c	1652c	Passage 16–19	Passage 24–26
GMO3349c	3349c	Passage 16–18	Passage 23–26
GMO5565	5565	Passage 14–20	Passage 25–28
GMO5757c	5757c	Passage 14–20	Passage 25–28
HGADFN003	P003	Passage 10–15	Passage 20–23
HGADFN127	P127	Passage 10–13	Passage 16–19
HGADFN164	P164	Passage 10–15	Passage 18–21
HGADFN178	P178	Passage 7–10	Passage 12–15
HGADFN188	P188	Passage 9–12	Passage 14–18
HGADFN271	P271	Passage 8–11	Passage 14–16
HGFDFN369	F369	Passage 10–14	Passage 16–18
HGMDFN368	M368	Passage 9–12	Passage 14–16

**Table 2:** Selected list of miR-181a targets or inducers based on literature search. Corresponding proteins are grouped according to their function.

Pathway	Protein	Abbreviation	Literature	Regulation
SASP	Interleukin 6	IL-6	[67]	Target
	Transforming growth factor β1	TGFβ1	[68]	Inducer
Energy metabolism	Sirtuin 1	SIRT1	[69]	Target
Cell survival	Phosphatase and tensin homolog	PTEN	[70]	Target
Autophagy	Autophagy-related protein 5	ATG5	[71]	Target
	Adenosine monophosphate-activated protein kinase	AMPK	[72]	Target
DNA damage	Ataxia-telangiectasia mutated	ATM	[68]	Target

## Data Availability

The datasets used and analyzed during the current study are available from the corresponding author on reasonable request.

## Supplementary Materials

The following supporting information can be downloaded at: https://www.mdpi.com/article/10.3390/diseases13080245/s1, Figure S1. miR-181a-5p regulates p21 expression. Figure S2. Full-length scans of Western Blots and Blots used for Quantification. Figure S3. Full-length scans of Western blots. Table S1: Stem-loop Reverse Transcription Primers for human miRNAs designed with sRNAPrimerDB [42,43]. Table S2. Primers for quantitative Polymerase Chain Reaction of miRNAs from fibroblast cultures designed with sRNAPrimerDB [43]. Table S3. Primers for quantitative Polymerase Chain Reaction of mRNAs from fibroblast cultures [24,66,96,97,98,99]. Table S4. Primers for quantitative Polymerase Chain Reaction for miRNAs and mRNAs isolated from mouse skin tissue [100,101].

## Abbreviations

The following abbreviations are used in this manuscript:

AMPK	5′-AMP-activated protein kinase catalytic subunit alpha-1
ATG5	Autophagy-related protein 5
ATM	Ataxia-telangiectasia mutated
ATP	Adenosine-Triphosphate
CCL2	C-C motif chemokine ligand 2
cGAS	Cyclic GMP-AMP
GAPDH	Glycerin-aldehyde-3-phosphate
HGPS	Hutchinson–Gilford progeria syndrome
IL-6/IL-1	Interleukin 6/1
JAK	Janus Kinase
microRNAs	miRNAs/miR
mTOR	Mechanistic target of rapamycin
NF-ĸB	Nuclear factor ‘kappa-light-chain-enhancer’ of activated B-cells
PTEN	Phosphatase and tensin homolog
RNU-6	U6 small nuclear 1
ROS	Reactive oxygen species
SASP	Senescence-associated secretory phenotype
SIRT1	Sirtuin 1
STAT	Signal transducer and activator of transcription
STING	Stimulator of interferon genes
TGFβ1	Transforming growth factor β1
TLR	Toll-like receptor
TNF	Tumor necrosis factor
TRAF	TNF receptor-associated factor
ULK1	Unc-51-like kinase
UTR	Untranslated region
