# Supplementary material for: Impact of miR-181a on SIRT1 Expression and Senescence in Hutchinson–Gilford Progeria Syndrome
Source: Diseases. 2025 Aug 4;13(8):245. doi: 10.3390/diseases13080245 (PMC12386028; doi:10.3390/diseases13080245)
Supplement: Supplementary file 1 [file diseases-13-00245-s001.zip › diseases-3728807-supplementary.pdf]

Impact of miR-181a on SIRT1 expression and senescence in Hutchinson-Gilford Progeria Syndrome.

Eva-Maria Lederer, Felix Quirin Fenzl, Peter Krüger, Moritz Schroll, Ramona Hartinger and Karima Djabali

Epigenetics of Aging, Department of Dermatology and Allergy, TUM School of Medicine, Technical University of Munich (TUM), 85748 Garching, Germany
* Correspondence: djabali@tum.de

Supplementary Material

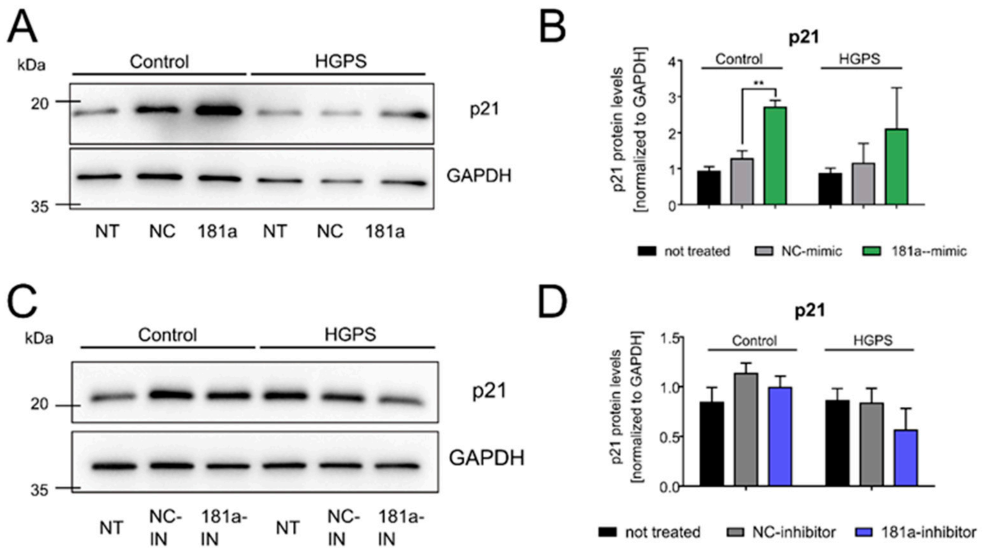

Figure S1. miR-181a-5p affects p21 expression. **(A)** Representative Western Blot analysis of p21 protein expression in control (HGFDFN369) and HGPS (HGADFN003) fibroblasts in comparison to GAPDH during miR-181a-5p mimic treatment. **(B)** Quantification of p21 protein levels during miR-181a-5p mimic treatment. **(C)** Representative Western Blot analysis of p21 protein expression in control (HGFDFN369) and HGPS (HGADFN003) fibroblasts in comparison to GAPDH during miR-181a-5p inhibitor treatment. **(D)** Quantification of p21 protein levels during miR-181a-5p inhibitor treatment. **(B, D)** Graphs show mean values of 3 control cell lines (GMO5567A, HGFDFN369, HGMDFN368) and three HGPS cell lines (HGADFN003, HGADFN271, HGADFN164) +/- standard deviation. Statistical significance was calculated using ANOVA with Tukey's Post Hoc test (* $p < 0.05$, (n=3)).

Table S1. Stem-loop Reverse Transcription Primers for human miRNAs designed with sRNAPrimerDB [42,43].

Name	Sequence Stem Loop Primer
hsa-miR-15a-5p	GTCGTATCCAGTGCAGGGTCCGAGGTATTCGCACTGGATACGACCACAAACC
hsa-miR-16-5p	GTCGTATCCAGTGCAGGGTCCGAGGTATTCGCACTGGATACGACCGCCAATA
hsa-miR-17-5p	GTCGTATCCAGTGCAGGGTCCGAGGTATTCGCACTGGATACGACCTACCTGC
hsa-miR-20a-5p	GTCGTATCCAGTGCAGGGTCCGAGGTATTCGCACTGGATACGACCTACCTGC
hsa-miR-21-5p	GTCGTATCCAGTGCAGGGTCCGAGGTATTCGCACTGGATACGACTCAACATC
hsa-miR-23b-5p	GTCGTATCCAGTGCAGGGTCCGAGGTATTCGCACTGGATACGACAAATCAGC
hsa-miR-25-3p	GTCGTATCCAGTGCAGGGTCCGAGGTATTCGCACTGGATACGACTCAGACCG
hsa-miR-29a-5p	GTCGTATCCAGTGCAGGGTCCGAGGTATTCGCACTGGATACGACCTGAACAC

hsa-miR-30a-5p	GTCGTATCCAGTGCAGGGTCCGAGGTATTCGCACTGGATACGACCTTCCAGT
hsa-miR-34a-5p	GTCGTATCCAGTGCAGGGTCCGAGGTATTCGCACTGGATACGACACAACCAG
hsa-miR-93-5p	GTCGTATCCAGTGCAGGGTCCGAGGTATTCGCACTGGATACGACCTACCT
hsa-miR-100-5p	GTCGTATCCAGTGCAGGGTCCGAGGTATTCGCACTGGATACGACCACAAGTT
hsa-miR-103a-3p	GTCGTATCCAGTGCAGGGTCCGAGGTATTCGCACTGGATACGACCAAGGC
hsa-miR-106b-5p	GTCGTATCCAGTGCAGGGTCCGAGGTATTCGCACTGGATACGACATCTGCAC
hsa-miR-125b-5p	GTCGTATCCAGTGCAGGGTCCGAGGTATTCGCACTGGATACGACTCACAAGT
hsa-miR-145-5p	GTCGTATCCAGTGCAGGGTCCGAGGTATTCGCACTGGATACGACAGGGATTC
hsa-miR-155-5p	GTCGTATCCAGTGCAGGGTCCGAGGTATTCGCACTGGATACGACAACCCC
hsa-miR-181a-5p	GTCGTATCCAGTGCAGGGTCCGAGGTATTCGCACTGGATACGACACTCACCG
hsa-miR-182-5p	GTCGTATCCAGTGCAGGGTCCGAGGTATTCGCACTGGATACGACAGTGTGAG
hsa-miR-191-5p	GTCGTATCCAGTGCAGGGTCCGAGGTATTCGCACTGGATACGACCAGCTGCT
hsa-miR-199a-5p	GTCGTATCCAGTGCAGGGTCCGAGGTATTCGCACTGGATACGACGAACAGGT
hsa-miR-211-5p	GTCGTATCCAGTGCAGGGTCCGAGGTATTCGCACTGGATACGACAGGCGAAG
hsa-miR-218-5p	GTCGTATCCAGTGCAGGGTCCGAGGTATTCGCACTGGATACGACACATGGTT
hsa-miR-221-5p	GTCGTATCCAGTGCAGGGTCCGAGGTATTCGCACTGGATACGACAAATCTAC
hsa-miR-222-5p	GTCGTATCCAGTGCAGGGTCCGAGGTATTCGCACTGGATACGACAGGATCTA
hsa-miR-361-3p	GTCGTATCCAGTGCAGGGTCCGAGGTATTCGCACTGGATACGACGTACCCCT
hsa-miR-423-5p	GTCGTATCCAGTGCAGGGTCCGAGGTATTCGCACTGGATACGACAAAGTCTC
U6	GTCGTATCCAGTGCAGGGTCCGAGGTATTCGCACTGGATACGACAAAATA

Table S1. Primers for quantitative Polymerase Chain Reaction of miRNAs from fibroblast cultures designed with sRNAPrimerDB [43]..

Name	Sequence Forward Primer	Sequence Reverse Primer
hsa-miR-15a-5p	AAGAGCGTTAGCAGCACATAAT	
hsa-miR-16-5p	AACCGGTAGCAGCACGTAAA	
hsa-miR-17-5p	AACAGTGCAAAGTGCTTACAGT	
hsa-miR-20a-5p	AATCGGCGTAAAGTGCTTATAGT	
hsa-miR-21-5p	ACCGAGGTTAGCTTATCAGACT	
hsa-miR-23b-5p	AACAAGTGGGTTCCTGGCAT	
hsa-miR-25-3p	AACAGTGCATTGCACTTGTCT	
hsa-miR-29a-5p	CCACGGTCACTGATTTCTTTTG	
hsa-miR-30a-5p	AAGAGCGTTGTAAACATCCTCG	
hsa-miR-34a-5p	AACAGTGTGGCAGTGTCTTAG	
hsa-miR-93-5p	AACACGCCAAAGTGCTGTTC	
hsa-miR-100-5p	AACACGTGAACCCGTAGATCC	
hsa-miR-103a-3p	AACACGCGGCTTCTTTACAG	
hsa-miR-106b-5p	ACCACCGTAAAGTGCTGACA	GTCGTATCCAGTGCAGGGT
hsa-miR-125b-5p	AACAGTGTCCCTGAGACCCTA	
hsa-miR-145-5p	AACAAGGTCCAGTTTTCCCAG	
hsa-miR-155-5p	AACGCACTTAATGCTAATCGTGA	
hsa-miR-181a-5p	AACACGTGAACATTCAACGCT	
hsa-miR-182-5p	AACACGTGTTTGGCAATGGTAG	
hsa-miR-191-5p	AAGAAGACAACGGAATCCCAAA	
hsa-miR-199a-5p	AACAAGCCCAGTGTTCAGACT	
hsa-miR-211-5p	AACACGCTTCCCTTTGTCATC	
hsa-miR-218-5p	AACACGTGTTGTGCTTGATCT	
hsa-miR-221-5p	AAGAGCGTACCTGGCATACAAT	
hsa-miR-222-5p	AACAGTGCTCAGTAGCCAGT	
hsa-miR-361-3p	AATCGGCGTTATCAGAATCTCC	
hsa-miR-423-5p	AACAAGTGAGGGGCAGAGAG	
U6	CTCGCTTCGGCAGCACA	AACGCTTCACGAATTTGCGT

Table S2. Primers for quantitative Polymerase Chain Reaction of mRNAs from fibroblast cultures.

Name	Sequence Forward Primer	Sequence Reverse Primer	Origin
TGFβ1	CCCAGCATCTGCAAAGCTC	GTCAATGTACAGCTGCCGCA	[24]
ATG5	TGGGCCATCAATCGGAAACTC	TGCAGCCACAGGACGAAACAG	[96]
AMPK (PRKAA1)	TTGAAACCTGAAAATGTCCTGCT	GGTGAGCCACAACTTGTTCTT	[97]
PTEN	AGGGACGAACTGGTGTAATGA	CTGGTCCTTACTTCCCCATAGAA	[98]
SIRT1	TGCTGGCCTAATAGAGTGGCA	CTCAGCGCCATGGAAAATGT	[99]
IL-6	GGTACATCCTCGACGGCATCT	GTGCCTCTTTGCTGCTTTCAC	[66]
GAPDH	GTCTCCTCTGACTTCAACAGCG	ACCACCCTGTTGCTGTAGCCAA	designed

Table S3. Primers for quantitative Polymerase Chain Reaction for miRNAs and mRNAs isolated from mouse skin tissue.

Name	Sequence Forward Primer	Sequence Reverse Primer	Origin
miR-181a-5p_ mouse	same sequence as human primers		
U6	same sequence as human primers		
SIRT1_mouse	GCTGACGAC TTCGACGACG	TCGGTCAACAGGAGGTTGTCT	[100}
TGFB1_mouse	CTCCCGTGGCTTCTAGTGC	GCCGTTGAATTTGCCGTGAG	designed
GAPDH_mouse	TTGTTGCCATCAACGACCCC	GCCGTTGAATTTGCCGTGAG	[101]

A

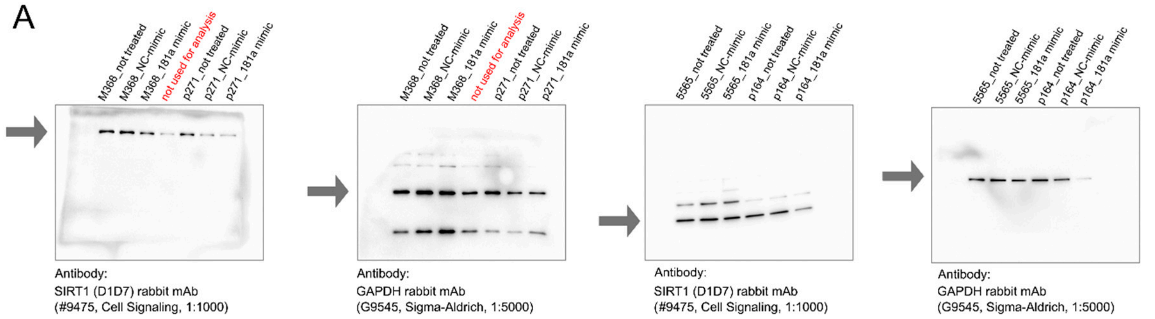

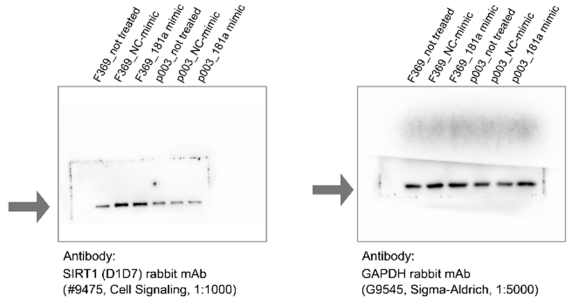

B

	not treated			negative control			miR-181a-5p		
Control	1	1,034807	1,332006	1,967173	1,283817	1,416734	1,564523	1,062717	0,844495
HGPS	1,040113	1,626921	1,25154	0,868143	0,952289	0,782781	0,506402	0,322195	0,369026

C

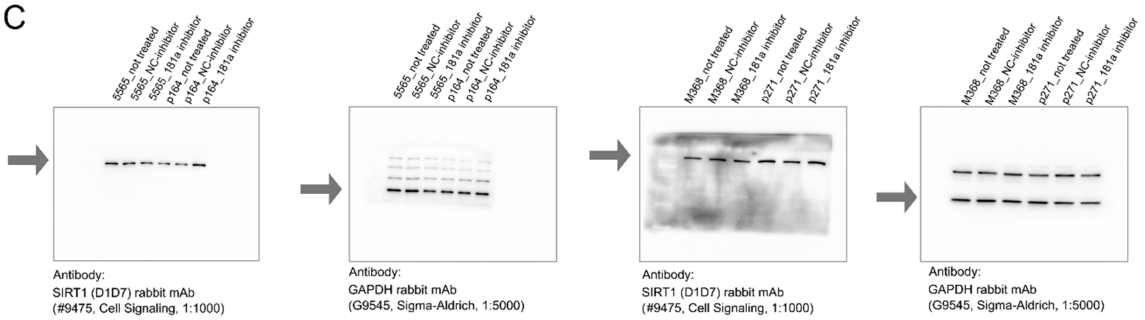

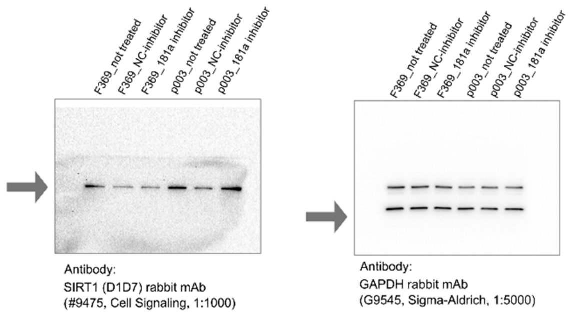

D

	not treated			negative control inhibitor			miR-181a-5p - inhibitor		
Control	1,192141	1	1,436829	0,897736	0,955352	0,567206	1,053598	1,146696	0,569899
HGPS	0,785822	1,677608	1.561517	0.912014	1.231212	0.756955	1.498776	1.968312	2,909991

Figure S2. Full-length scans of Western Blots and Blots used for Quantification. **(A)** Full-length scans of Western blots of SIRT1 quantification during miR-181a-5p mimic treatment and respective negative-control treatment. Analyzed lanes are marked with an arrow. **(B)** Quantification values normalized to GAPDH and referred to one control cell line. **(C)** Full-length scans of Western blots of SIRT1 quantification during miR-181a-5p inhibitor treatment and respective negative-control treatment. Analyzed lanes are marked with an arrow. **(D)** Quantification values normalized to GAPDH and referred to one control cell line.

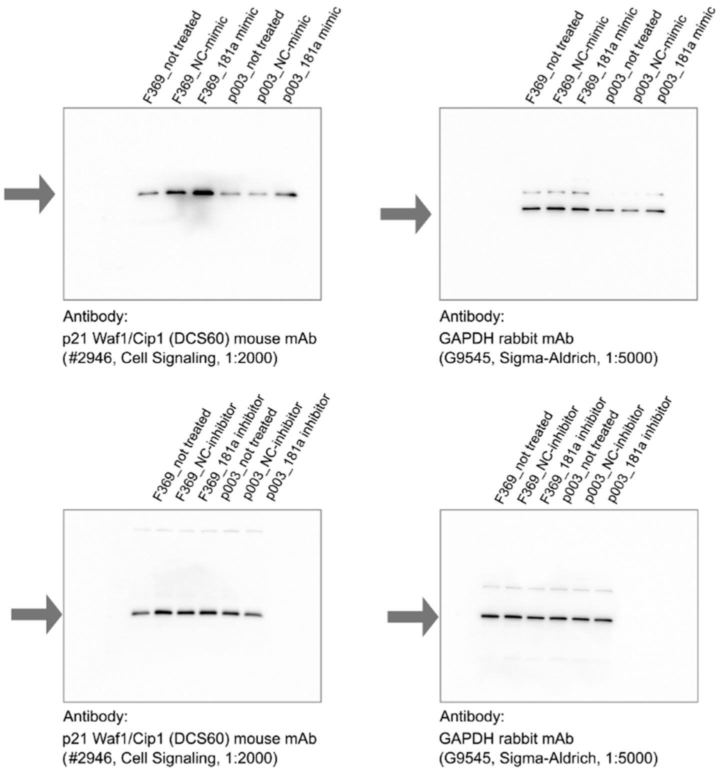

Figure S3. Full-length scans of Western blots. Raw data files for Western blots in Figure S1. Abbreviations: NC = negative control.

References

[24] Liu, C.; Arnold, R.; Henriques, G.; Djabali, K. Inhibition of JAK-STAT Signaling with Baricitinib Reduces Inflammation and Improves Cellular Homeostasis in Progeria Cells. *Cells*, **2019**, *8*.

[42] Chen, C.; Ridzon, D.A.; Broomer, A.J.; Zhou, Z.; Lee, D.H.; Nguyen, J.T.; Barbisin, M.; Xu, N.L.; Mahuvakar, V.R.; Andersen, M.R.; Lao, K.Q.; Livak, K.J.; Guegler, K.J. Real-time quantification of microRNAs by stem-loop RT-PCR. *Nucleic acids research*, **2005**, *33*, e179.

[43] Xie, S.; Zhu, Q.; Qu, W.; Xu, Z.; Liu, X.; Li, X.; Li, S.; Ma, W.; Miao, Y.; Zhang, L.; Du, X.; Dong, W.; Li, H.; Zhao, C.; Wang, Y.; Fang, Y.; Zhao, S. sRNAPrimerDB: comprehensive primer design and search web service for small non-coding RNAs. *Bioinformatics (Oxford, England)*, **2019**, *35*, 1566–1572.

[66] Arnold, R.; Vehns, E.; Randl, H.; Djabali, K. Baricitinib, a JAK-STAT Inhibitor, Reduces the Cellular Toxicity of the Farnesyltransferase Inhibitor Lonafarnib in Progeria Cells. *International journal of molecular sciences*, **2021**, *22*.

[96] Park, J.W.; Kim, Y.; Lee, S.-B.; Oh, C.W.; Lee, E.J.; Ko, J.Y.; Park, J.H. Autophagy inhibits cancer stemness in triple-negative breast cancer via miR-181a-mediated regulation of ATG5 and/or ATG2B. *Molecular oncology*, **2022**, *16*, 1857–1875.

[97] Zhang, Y.; Zhou, X.; Cheng, L.; Wang, X.; Zhang, Q.; Zhang, Y.; Sun, S. PRKAA1 Promotes Proliferation and Inhibits Apoptosis of Gastric Cancer Cells Through Activating JNK1 and Akt Pathways. *Oncology research*, **2020**, *28*, 213–223.

[98] Liu, H.-Y.; Zhang, Y.-Y.; Zhu, B.-L.; Feng, F.-Z.; Yan, H.; Zhang, H.-Y.; Zhou, B. miR-21 regulates the proliferation and apoptosis of ovarian cancer cells through PTEN/PI3K/AKT. *European review for medical and pharmacological sciences*, **2019**, *23*, 4149–4155.

[99] Yan, P.; Li, Z.; Xiong, J.; Geng, Z.; Wei, W.; Zhang, Y.; Wu, G.; Zhuang, T.; Tian, X.; Liu, Z.; Liu, J.; Sun, K.; Chen, F.; Zhang, Y.; Zeng, C.; Huang, Y.; Zhang, B. LARP7 ameliorates cellular senescence and aging by allosterically enhancing SIRT1 deacetylase activity. *Cell reports*, **2021**, *37,* 110038.

[100] Dang, R.; Wang, M.; Li, X.; Wang, H.; Liu, L.; Wu, Q.; Zhao, J.; Ji, P.; Zhong, L.; Licinio, J.; Xie, P. Edaravone ameliorates depressive and anxiety-like behaviors via Sirt1/Nrf2/HO-1/Gpx4 pathway. *Journal of neuroinflammation*, **2022**, *19,* 1–29.

[101] Lim, S.H.; Lee, H.S.; Han, H.-K.; Choi, C.-I. Saikosaponin A and D Inhibit Adipogenesis via the AMPK and MAPK Signaling Pathways in 3T3-L1 Adipocytes. *International journal of molecular sciences*, **2021**, *22*
